# Supplementary material for: Somatic mutagenesis in satellite cells associates with human skeletal muscle aging
Source: Nat Commun. 2018 Feb 23;9:800. doi: 10.1038/s41467-018-03244-6 (PMC5824957; doi:10.1038/s41467-018-03244-6)
Supplement: Supplementary file 1 — Supplementary Information [file 41467_2018_3244_MOESM1_ESM.pdf]

Supplementary Information

Somatic mutagenesis in satellite cells contributes to human skeletal muscle aging

Franco et al.

Supplementary Figure 1: FACS sorting of CD56-positive satellite cells from muscle biopsies

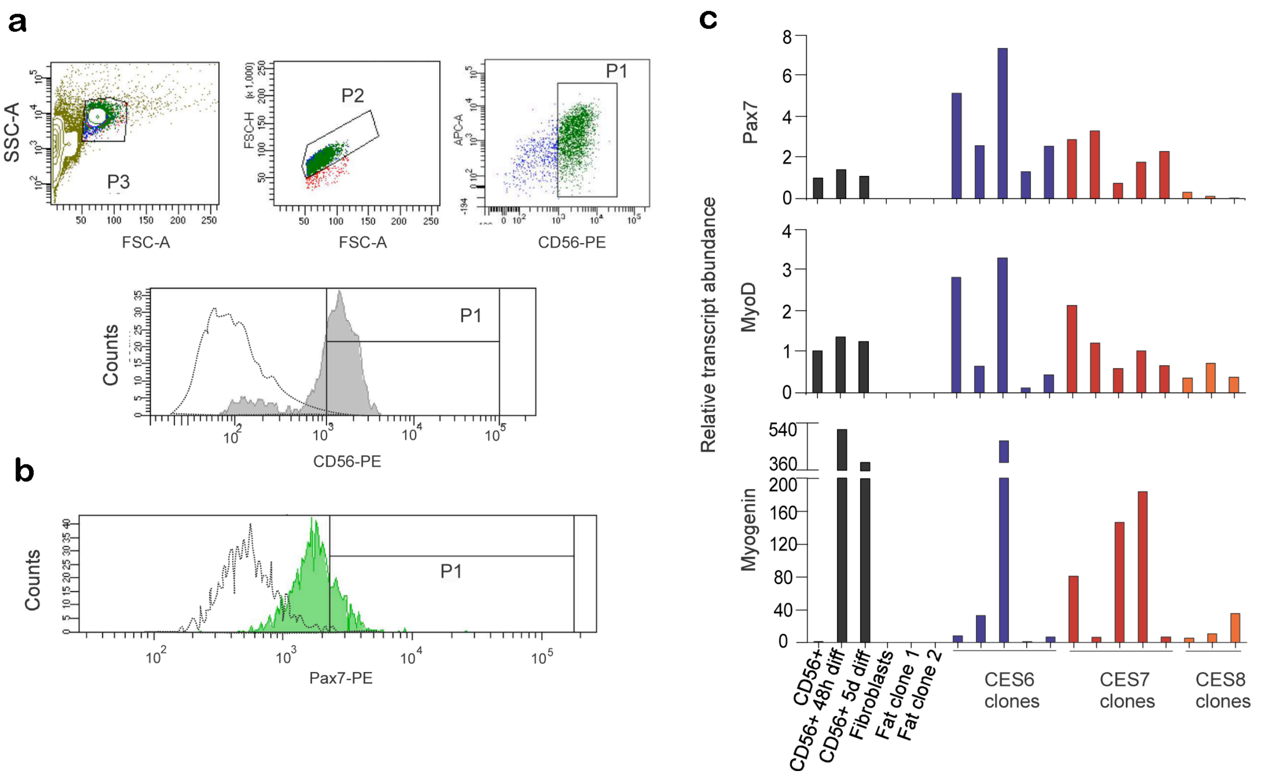

(a) An example of the sorting strategy used to obtain single satellite cell clones (SCCs) from human biopsies of the *vastus lateralis* muscle. Freshly isolated cells were left in a cell culture dish for 48 h. An SC-enriched population of adherent cells was detached and stained with the SC marker CD56. Since this antigen can also be found on different types of human blood cells, a gate based on cell size (FSC) and granularity (SSC) was set (P3) in order to exclude contaminant cells with high size and granularity (granulocytes and monocytes). The cells were also sorted based on cell size for doublet discrimination (P2). Finally, the cells that stained positive for CD56-PE were sorted (P1). The antigen expression was compared to the ISO-IgG staining (histogram, dashed line). The cells gated in P1 were 60.3±4.3% of the cells from the parental population (P2), n=7 biopsies. (**b**) Representative FACS analysis of Pax7 expression in an expanded $CD56^+$ cell population used in the study (biopsy CES3). Unsorted cells left from the single cell sorting were expanded in vitro for 4 weeks, then sorted for CD56 and tested for the expression of Pax7. Comparison with cells stained with the secondary Ab only (dashed line) showed a uniform and weak expression of the antigen. The gate P1 was used to quantify the percentage of cells expressing detectable levels of Pax7 for the different $CD56^+$ populations shown in supplementary table 1. (**c**) QPCR analysis of the myogenic markers Pax7, MyoD and myogenin in sequenced clones from biopsies CES6, CES7 and CES8. As a positive control, the $CD56^+$ population showed in **b** was used. RNA was extracted from cells either kept in normal growing conditions ($CD56^+$) or in differentiating medium (for 48 hours or 5 days). Negative controls were a population of human fibroblasts and two different human clones from fat progenitors. While in the negative controls myogenic genes were completely silenced, all the SCCs showed Pax7, MyoD and myogenin expression, thus confirming their satellite cell origin.

Supplementary Figure 2: SCC substitution profiles.

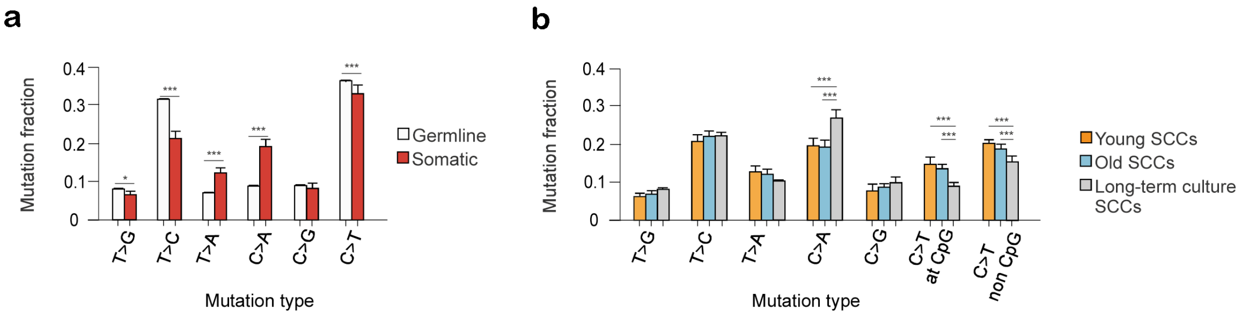

(a) Substitution spectrum for germline (SNVs found in blood and muscle bulk samples from all individuals included in the study) and somatic variants (all SCCs). *P<0.05 ***P<0.001; two-way ANOVA, multiple comparison test. (**b**) Substitution spectrum in young, old and long-culture SCCs. Young and old SCCs appear very similar. In contrast, long-term culture SCCs, which were cultured for 50 days as a population before the clonal culture, showed increased C>A and decreased C>T conversions, possibly representing culture induced mutations. ***P<0.001; two-way ANOVA, multiple comparison test.

Supplementary Figure 3: *De novo*-extracted mutational signatures in SCCs.

a

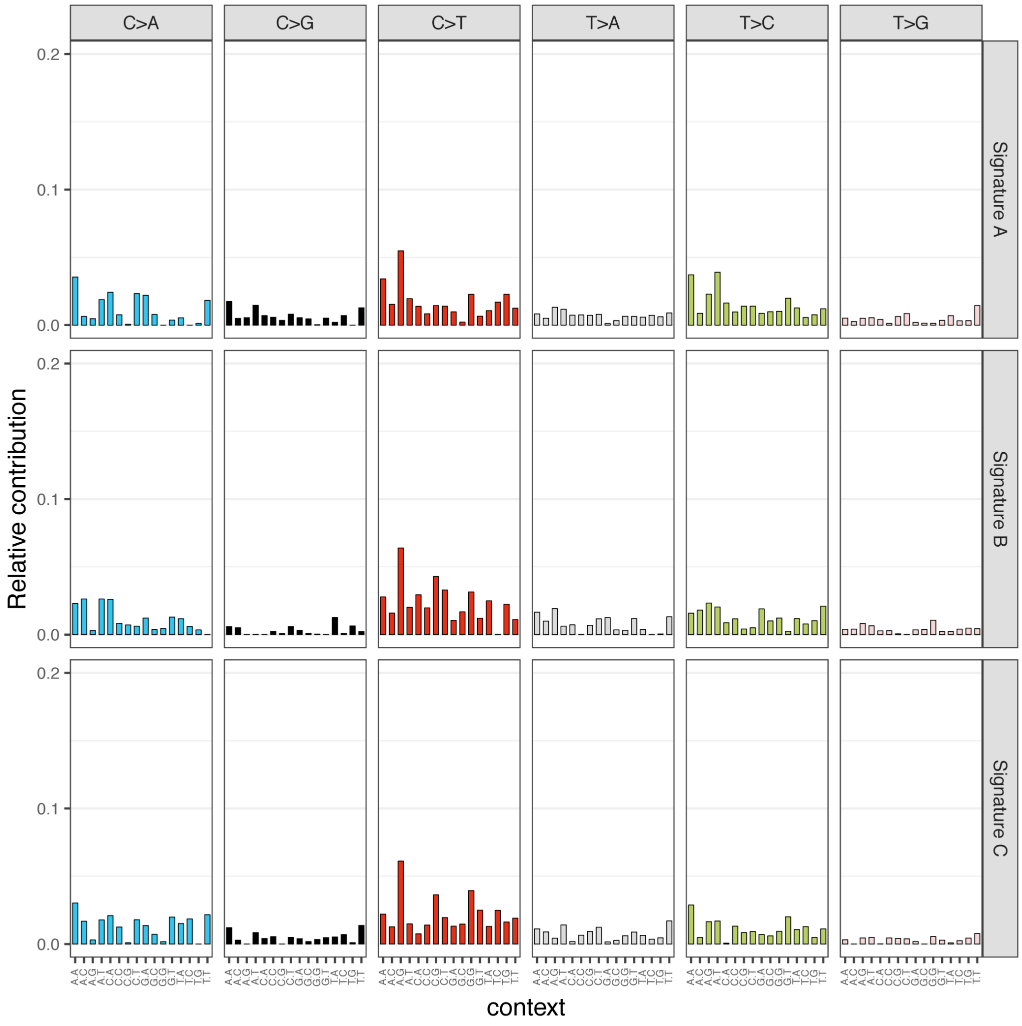

b

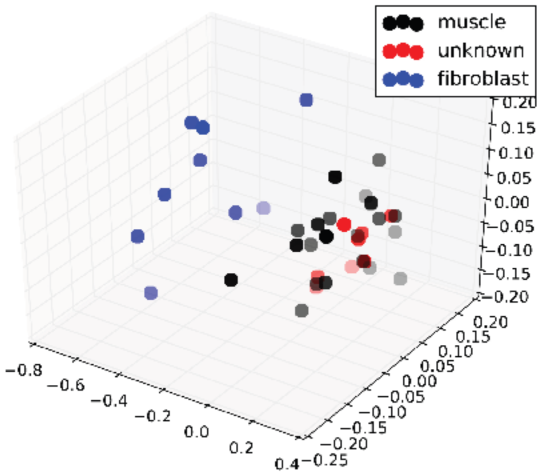

(a) Lists of SNVs in SCCs cultured with the standard protocol (N=29) were used to define the satellite cell-specific mutation signatures named A, B and C, using the MutationalPatterns tool[1]. The signatures are based on a classification of substitutions refined by the inclusion of the 5' and 3' sequence context of each mutated nucleotide to obtain 96 mutation classes[2] (bottom). (**b)** Principal component analysis of mutational signatures extracted from 29 SCCs and fibroblast clones from[3]. An homogenous mutational signature clusters all the 29 sequenced SSCs (muscle and unknown), indicating that all 29 clones are derived from satellite cells.

Supplementary Figure 4: Contribution of COSMIC signatures to the SCC mutation catalogues

a

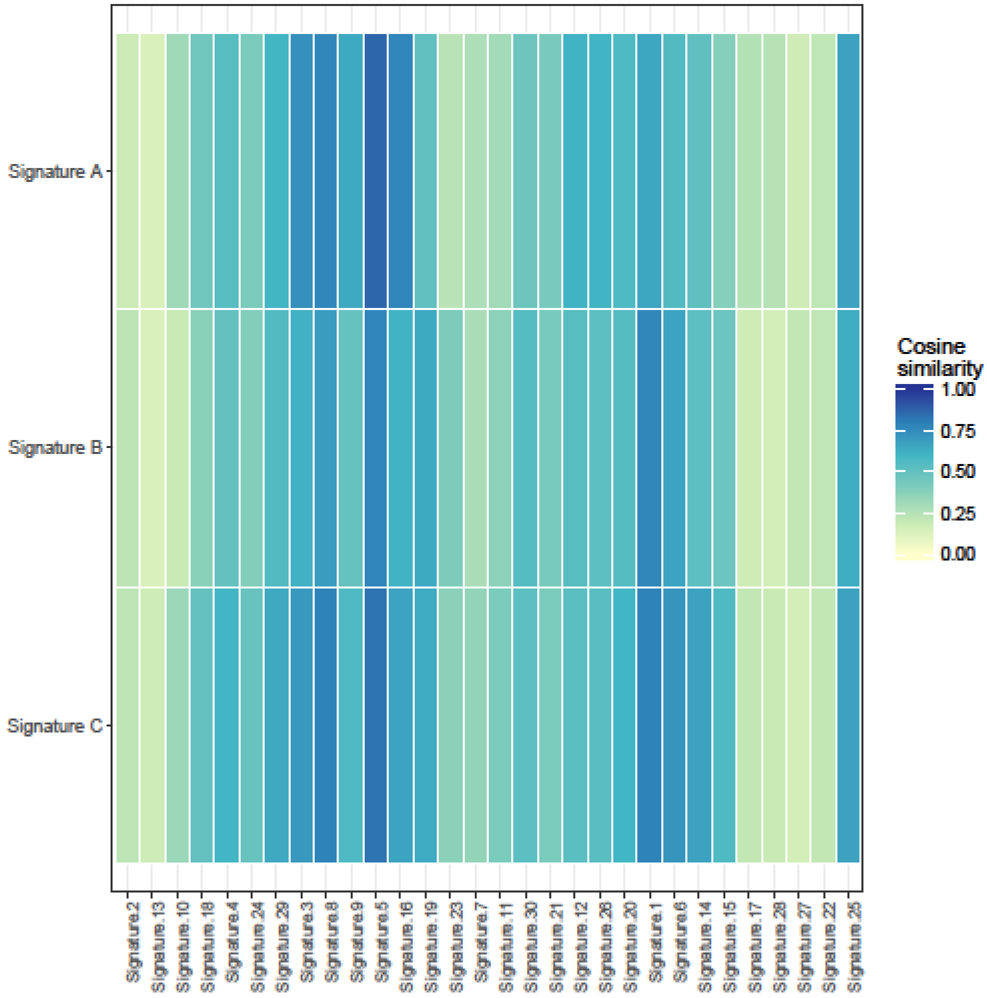

b

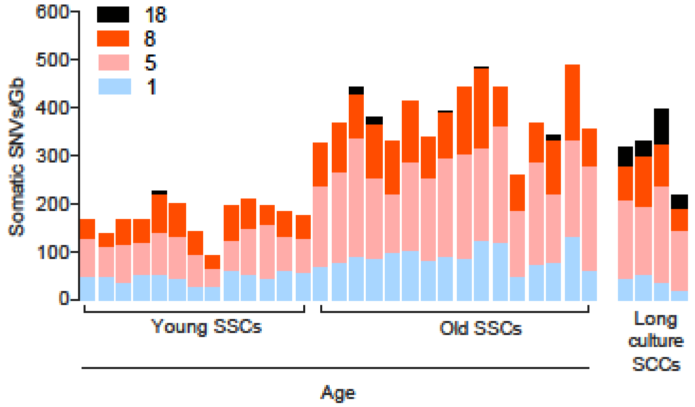

(a) Cosine similarity of signatures A, B, C (*de novo*-extracted from the 29 SCCs, Supplementary Figure 3) with the cancer signatures classified in http://cancer.sanger.ac.uk/cosmic/signatures (COSMIC signatures). Best three results for signature A: signature 5 (cosine similarity: 0.865), 8 (cosine

similarity: 0.776) and 16 (cosine similarity: 0.770); signature B: signature 5 (cosine similarity: 0.773), 1 (cosine similarity: 0.769) and 8 (cosine similarity: 0.695); signature C: signature 5 (cosine similarity: 0.832), 8 (cosine similarity: 0.785) and 1 (cosine similarity: 0.785). **(b)** Absolute contribution of signatures 1, 5, 8 and 18 to the mutation catalogue of young, old and long-culture SCCs. Signature 18 is almost exclusively found in long-culture SCCs, in agreement with its occurrence during extended cell culture.

Supplementary Figure 5: Genomic distribution of somatic mutations in young and old clones

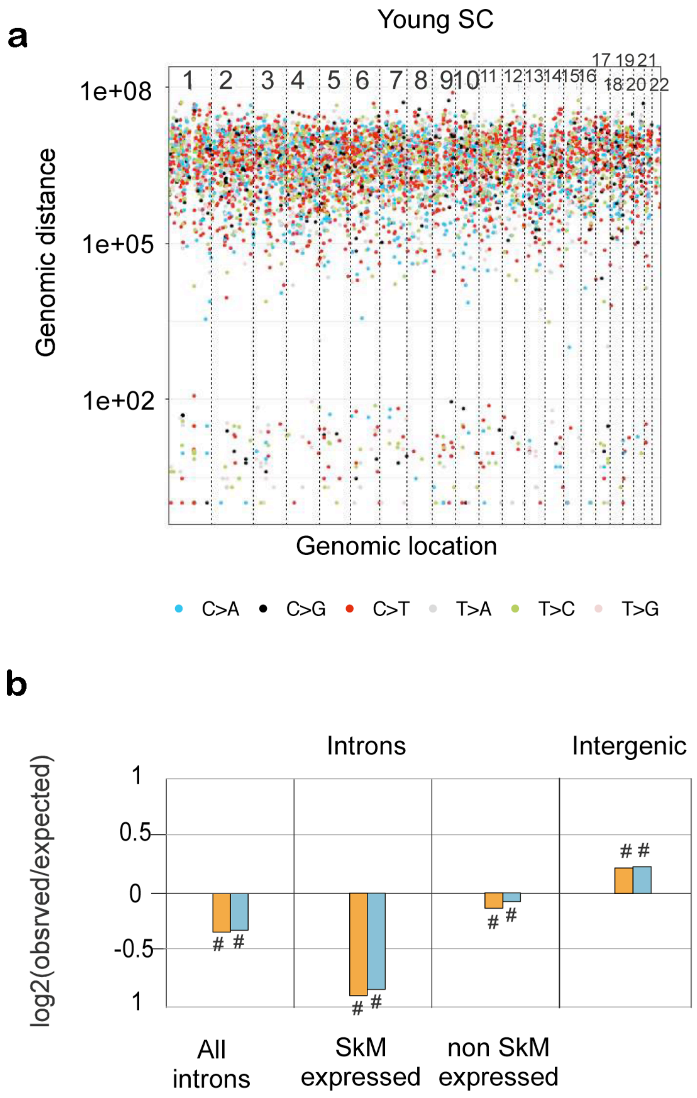

(a) Rainfall plots of all somatic SNVs detected in the young and old SCCs showing the distribution of the mutations across chromosomes (*x*-axis, top) and the distance of each mutation from the closest one (genomic distance, *y*-axis) expressed as the number of bases. Different dot colors correspond to the different substitution types. (**b**) The $\log_2$ ratio of the number of observed and expected point mutations indicating the effect size of the enrichment or depletion in intronic (expressed or non expressed in the skeletal muscle, SkM) and intergenic regions. #*P*<0.05, one-sided binomial test.

Supplementary Figure 6: Transcribed regions during myoblast to myotube differentiation.

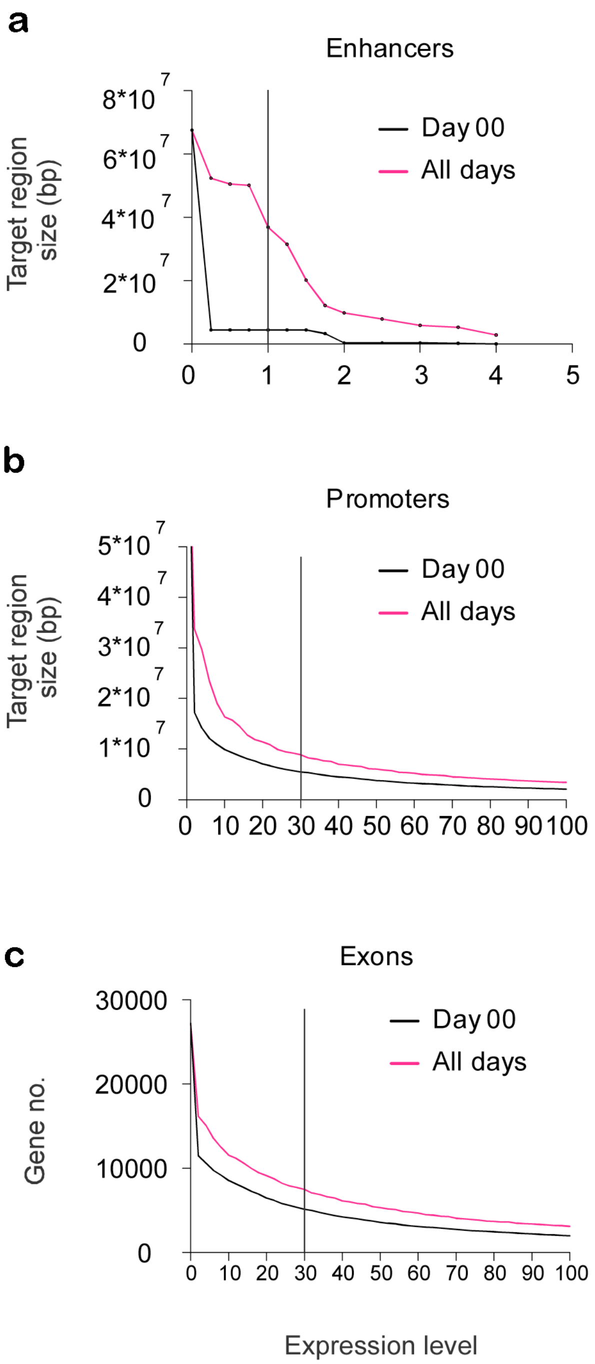

Threshold used to define the expressed enhancers, promoters and genes obtained from the "Myoblast differentiation to myotube" dataset from the FANTOM5 project http://fantom.gsc.riken.jp/data/. The expression data were available at 8 different time points during the myoblast to myotube differentiation (CNhs13847-14585), as follows: day 00 (basal); day 01, day 02, day 03, day 04, day 06, day 08, day 10, and day 12 following exposure to the differentiation stimuli. The expression levels before (day 0)

and after the induction of the differentiation were separately analyzed. (**a)** The size of the genomic region considered "expressed enhancers" on day 0 (basal) or at all time points is shown as a function of the enhancer expression levels. In this study, only enhancers expressed with score ≥1 were included. The threshold is shown as a vertical bar. (**b)** For the promoters, the size of the expressed regions is shown as a function of the expression levels. The threshold was set as ≥30. (**c)** For the exons, the gene names were derived from the expressed regions that were included in the promoter list, and the number of genes as a function of the expression levels is shown.

Supplementary Figure 7: Rare event detection ddPCR analysis of variant c.7825C>T in gene *HSPG2* in SCC, blood and skeletal muscle samples.

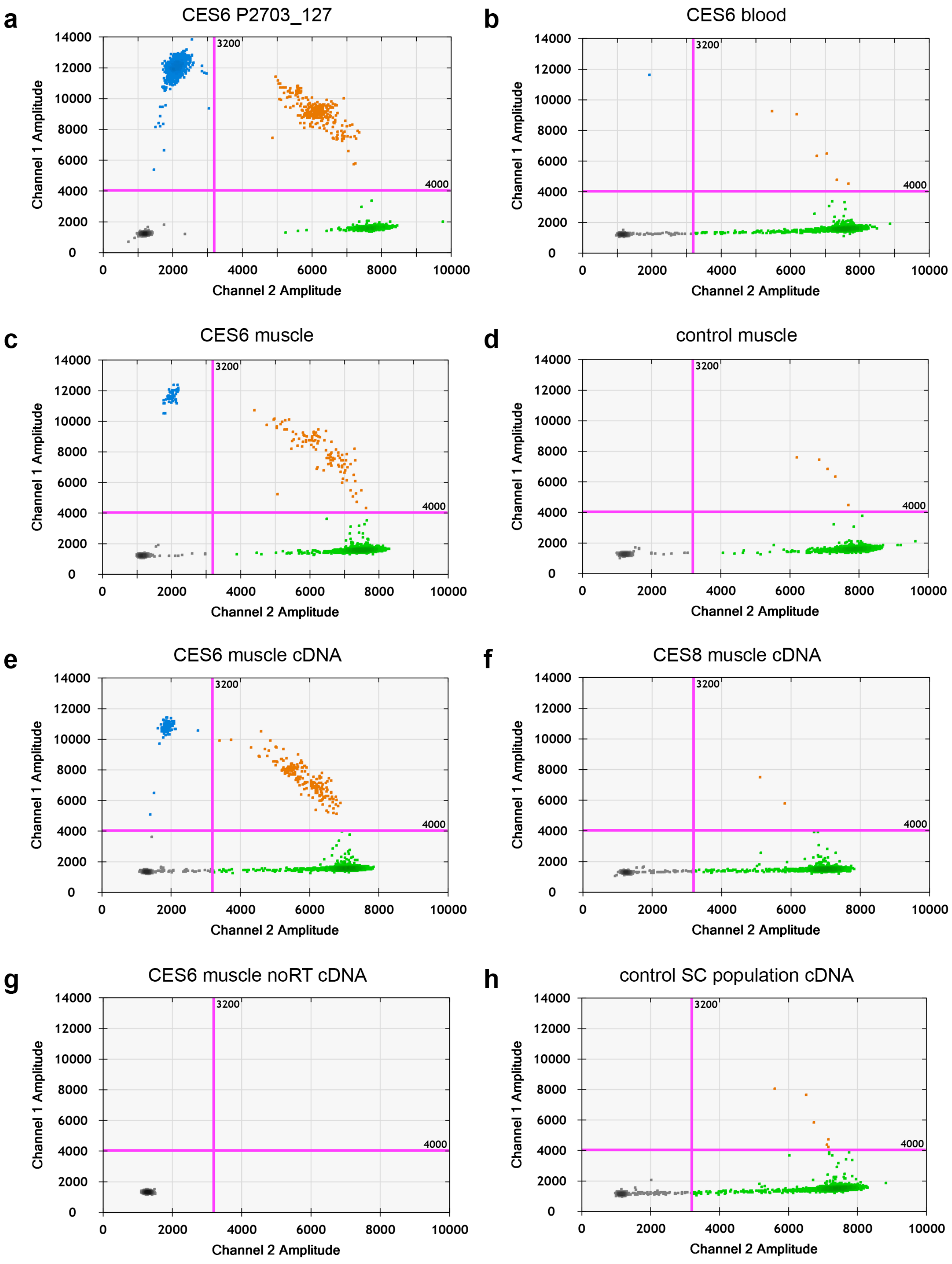

Representative examples of 2D plots from the rare event detection ddPCR analysis of variant c.7825C>T in gene *HSPG2* in satellite cell clone CES6 P2703_127 DNA **(a)**, CES6 blood DNA **(b)**, CES6 skeletal muscle DNA **(c)**, control skeletal muscle DNA **(d)**, CES6 skeletal muscle cDNA **(e)**, control skeletal muscle cDNA **(f)**, CES6 skeletal muscle noRT control **(g)** and control satellite cell population cDNA **(h)**. Channel 1 fluorescence (mutant probe labeled with FAM) is plotted against channel 2 fluorescence (wild-type probe labeled with HEX). Blue dots represent droplets containing only mutant alleles, orange dots represent droplets containing both wild-type and mutant alleles, green dots represent droplets containing only wild-type alleles, while gray dots represent empty droplets. SCC: satellite cell clone, SkM: skeletal muscle, control SkM: skeletal muscle biopsy from unrelated individual.

Supplementary Note 1: List of tested genes associated with muscle diseases.

ABHD5, ACADVL, ACTA1, ACVR1, AGL, AGRN, ALG14, ALG2, ANO5, ATP2A1, B3GALNT2, B3GNT1, B3GNT2, BAG3, BIN1, C10orf2, CACNA1A, CACNA1S, CAPN3, CASQ1, CAV3, CFL2, CHAT, CHCHD10, CHKB, CHRNA1, CHRNB1, CHRND, CHRNE, CHRNG, CLCN1, CLN3, CNBP, CNTN1, COL6A1, COL6A2, COL6A3, COLQ, CPT2, CRYAB, DAG1, DES, DMPK, DNAJB6, DNM2, DOK7, DPAGT1, DPM1, DPM2, DPM3, DUX4, DYSF, ENO3, ETFA, ETFB, ETFDH, FKRP, FKTN, FLNC, GAA, GBE1, GFPT1, GMPPB, GNE, GYG1, GYS1, HNRNPDL, HSPG2, IKBKAP, ISCU, ISPD, ITGA7, KBTBD13, KCNA1, KCNE1, KCNE2, KCNE3, KCNH2, KCNJ18, KCNJ2, KCNQ1, KIF21A, KLHL40, KLHL41, KLHL9, LAMA2, LAMB2, LARGE, LDB3, LDHA, LIMS2, LMNA, LMOD3, LPIN1, MATR3, MEGF10, MSTN, MUSK, MYBPC3, MYH2, MYH3, MYH7, MYH8, MYOT, NEB, OPA1, ORAI1, PABPN1, PFKM, PGAM2, PGM1, PHOX2A, PLEC, PNPLA2, PNPLA8, POLG, POLG2, POMGNT1, POMGNT2, POMK, POMT1, POMT2, PREPL, PRKAG2, PTPLA, PTRF, PUS1, PYGM, RAPSN, RBCK1, RRM2B, RYR1, SCN4A, SCN5A, SEPN1, SGCA, SGCB, SGCD, SGCE, SGCG, SLC22A5, SLC25A20, SLC25A4, SMCHD1, SPEG, STIM1, SUCLA2, SYNE1, SYNE2, SYT2, TCAP, TIA1, TK2, TMEM43, TMEM5, TNNI2, TNNT1, TNNT3, TNPO3, TOR1A, TOR1AIP1, TPM2, TPM3, TRAPPC11, TRAPPC11, TRIM32, TRIM54, TRIM63, TTN, TTR, TUBB3, VCP, YARS2

The list of genes responsible for muscle diseases was adopted from the gene table of monogenic neuromuscular disorders (nuclear genome), which is updated yearly and available at www.musclegenetable.fr. Only disease groups associated with myocyte dysfunction were selected, while genes associated with neuron or cardiomyocyte function were excluded. Genes are shown in alphabetic order.

Supplementary Table 1. Satellite cell purity of $CD56^+$ cells isolated from the biopsies used in the study

	freshly isolated cells					expanded populations				
biopsy	% $CD56^+$ cells	no. sorted CD56+ plated as single cells	no. clones expressing MyoD	no. sorted $CD56^+$ plated as population	Pax7 transcript levels	% $CD56^+$ cells	% $CD56^+$ positive for Pax7	% $CD56^+$ positive for CD45	% $CD56^+$ positive for TE7	no. clones expressing MyoD
CES1	63.5	96	3/3	0	RNA n.a.	97.7	20.1	3.6	0	n.a.
CES2	80.1	144	n.a.	1562	2.36	n.a.	n.a.	n.a.	n.a.	n.a.
CES3	66.4	144	n.a.	50	RNA n.a.	97.5	22.5	0.5	4.1	4/4
CES4	51.2	144	n.a.	500	2.74	n.a.	n.a.	n.a.	n.a	n.a.
CES6	51.2	144	8/9	80	3.17	91.0	3	3	2.4	3/3
CES7	47.2	96	14/14	99	2.91	94.2	6.9	0.6	0	6/6
CES8	62.5	96	13/13	0	RNA n.a.	96.0	24	0.2	0.3	6/6

The characterization of the satellite cell purity in the $CD56^+$ sorted populations obtained from every biopsy used in the study was performed either in parallel with the original cell sorting (freshly isolated cells, left part of the table) or using the unsorted cells left by the sorting and expanded in vitro for 4 weeks (expanded populations, right part of the table)**.** The expression of MyoD was measured by qPCR. The transcript levels of Pax7 were measured by qPCR in the sorted $CD56^+$ cells plated as a population and expanded for 1 week. Values are shown as a relative expression, using the CES3 expanded population as a reference (see Supplementary Fig. 1b). The % of Pax7 and CD45 (bone-marrow derived) positive cells was assessed by FACS. The % of TE7 (fibroblast) cells was assessed by immunofluorescence on cytospun cells.

Supplementary Table 2. Sequenced SCCs, coverage and somatic mutations.

individual	clone name	age	myogenic origin verificatio n	coverag e	SNVs	indels	normalized SNVs	normalized indels
CES1	P2703_101	24	myotubes	0.85	536	29	627.9	34.0
	P2703_102	24	myotubes	0.85	372	26	438.5	30.6
	P2703_103	24	myotubes	0.74	209	8	281.3	10.8
	P2703_104	24	myotubes	0.92	558	37	607.8	40.3
	P2703_105	24	myotubes	0.93	585	32	632.4	34.6
CES7	P2703_131	21	Pax7, MyoD myogenin	0.91	478	46	523.6	50.4
	P2703_132	21	Pax7, MyoD myogenin	0.91	385	37	425.3	40.9
	P2703_133	21	Pax7, MyoD myogenin	0.88	467	39	528.9	44.2
	P2703_134	21	Pax7, MyoD myogenin	0.89	456	28	509.9	31.3
	P2703_135	21	Pax7, MyoD myogenin	0.92	623	44	678.8	47.9
CES8	P5364_103	24	Pax7, MyoD myogenin	0.92	579	36	632.5	39.3
	P5364_104	24	Pax7, MyoD myogenin	0.93	523	43	561.1	46.1
	P5364_105	24	Pax7, MyoD myogenin	0.93	485	35	521.0	37.6
CES2	P2703_109	75	n.a.	0.92	1362	83	1480.4	90.2
	P4206_203	75	n.a.	0.92	1265	69	1369.8	74.7
CES3	P2703_111	63	n.a.	0.88	869	67	991.1	76.4
	P2703_112	63	myuotube s	0.92	1031	84	1118.1	91.1
	P2703_113	63	myotubes	0.93	1264	82	1362.5	88.4
	P2703_114	63	n.a.	0.92	1063	66	1161.3	72.1
	P2703_115	63	n.a.	0.92	952	60	1034.7	65.2

individual	clone name	age	myogenic origin verification	coverage	SNVs	indels	normalized SNVs	normalized indels
CES4	P2703_116	67	n.a.	0.91	1146	66	1255.3	72.3
	P2703_117	67	n.a.	0.90	920	64	1018.6	70.9
	P2703_118	67	myotubes	0.92	1127	70	1229.5	76.4
	P2703_120	67	n.a.	0.93	1248	59	1347.4	63.7
CES6	P2703_126	78	Pax7, MyoD myogenin	0.80	641	47	801.7	58.8
	P2703_127	78	Pax7, MyoD myogenin	0.91	1019	74	1118.7	81.2
	P2703_128	78	Pax7, MyoD myogenin	0.74	765	48	1034.4	64.9
	P2703_129	78	Pax7, MyoD myogenin	0.93	1371	109	1481.8	117.8
	P2703_130	78	Pax7, MyoD myogenin	0.91	980	65	1081.6	71.7
long term culture	P2703_106	26	n.a.	0.90	868	52	967.4	58.0
	P2703_107	26	n.a.	0.92	971	92	1054.4	99.9
	P2703_108	26	n.a.	0.91	1089	74	1194.8	81.2
	P2703_110	26	n.a.	0.84	551	34	654.4	40.4

Satellite cell clones (SCCs) sequenced in the study were derived from 7 donors (first column) of different ages. The myogenic origin of 21/29 SCCs was tested with either a differentiation assay (myotubes) or qPCR for the expression of the myogenic genes Pax7, MyoD and myogenin. All tested clones resulted positive. Coverage indicates the percentage of autosomes covered by at least 15 reads in each sample. SNVs: single nucleotide variants. Indels: insertion deletions. SNVs and indels were normalized on coverage (last columns).

Supplementary Table 3: Estimate of false negative rate using clones and blood from the CES6 sample.

Sample	Germline heterozygotes with depth > 15X in clone	Called in clone by GATK and/or Fermikit and/or MuTect	0.4 < AF < 0.6 (AF=allele frequency in mpileup)	Filter 1 positive prediction rate	Number of high confidence germline homozygote SNVs	Minor allele frequency < 0.1	Filter 2 positive prediction rate	False negative rate
P2703_127	1270776	1252596	729115	0.573755721	n/a	n/a	n/a	0.43162001
P2703_129	1293526	1278777	818920	0.633091256	n/a	n/a	n/a	0.372840411
P2703_130	1262696	1247062	723616	0.57307222	n/a	n/a	n/a	0.432297106
CES6 blood	n/a	n/a	n/a	n/a	1341392	1328824	0.990630628	n/a
Average:				0.593306399				0.412252509

Supplementary Table 4. Validation of variants through technical replicates of sequencing

discovery set (SCC)	validation set (SCC)	n tested SNVs	% validated SNVs	n tested INDELs	% validated INDELs
P2703_113	P2703_113b	798	**93.73**	82	**84.15**
P2703_113	P2703_111	1222	4.26	80	8.75
P2703_113	P2703_112	1254	4.23	82	8.54
P2703_113	P2703_114	1247	3.53	83	7.23
P2703_113	P2703_115	1256	4.54	85	8.24
P2703_113	P2703_116	1247	4.33	83	7.23
P2703_113	P2703_117	1236	3.56	80	5.00
P2703_113	P2703_118	1250	4.32	81	6.17
P2703_113	P2703_119	1245	3.37	84	4.76
P2703_113	P2703_120	1258	3.58	84	4.76
P2703_116	P2703_119	1140	**98.77**	66	**98.48**
P2703_116	P2703_111	1132	4.06	64	1.56
P2703_116	P2703_112	1141	4.21	67	2.99
P2703_116	P2703_113	1146	5.24	66	1.52
P2703_116	P2703_114	1137	4.31	66	1.52
P2703_116	P2703_115	1144	4.72	67	2.99
P2703_116	P2703_117	1132	5.04	63	3.17
P2703_116	P2703_118	1136	5.72	67	1.49
P2703_116	P2703_120	1142	5.52	67	2.99

To validate somatic variants found in the study, 2 SCCs (P2703_113 and P2703_116) were subjected to a second round of whole genome sequencing (validation set), with independent library preparation. Clone P2703_113 was sequenced twice with independent library preparations. Clone P2703_116 was split into 2 wells during cell culture (1000 cell-stage) and resulted in 2 independently grown clones (P2703_116 and P2703_119) derived from the same ancestor cell. Validation was also tested in unrelated SCCs (P2703_111/120) to test the unspecific background signal of the method when comparing 2 random samples. Variants were called with our pipeline in the discovery set and then tested in the validation set. Variants were considered validated when present with a minimum coverage of 15x and a minimum of 3 reads supporting the alternative allele.

Supplementary Table 5. Validation of SNVs through sequenome MALDI-TOF and ddPCR

individual	clone	variant	gene	method
CES2	P2703_109	7_150761675_C/T	SLC4A2	Sequenom
CES3	P2703_111	17:79514999 C/T	C17orf70	Sequenom
CES3	P2703_114	12:1988201 C/T	CACNA2D4	Sequenom
CES3	P2703_113	1:34330303 C/T	HMGB4	Sequenom
CES3	P2703_113	4:99338610 C/T	RAP1GDS1	ddPCR
CES3	P2703_113	3:50426879 C/T	CACNA2D2	ddPCR
CES3	P2703_111	2:179560728 G/A	TTN	ddPCR
CES4	P2703_117	4:46976385 A/T	GABRA4	Sequenom
CES4	P2703_120	3_44762365_G/C	ZNF502	Sequenom
CES4	P2703_120	2:216272892 C/T	FN1	ddPCR
CES6	P2703_129	2:186660905 T/A	FSIP2	Sequenom
CES6	P2703_127	1:22174499 G/A	HSPG2	Sequenom and ddPCR
CES6	P2703_127	19:36339677 A/T	NPHS1	Sequenom
CES6	P2703_128	3:122630767 C/T	SEMA5B	Sequenom
CES6	P2703_129	6:159682281 T/A	FNDC1	ddPCR
CES7	P2703_133	4:100571122 G/A	RP11-766F14_2	Sequenom

Variants with predicted high impact on the encoded protein were selected across all clones. 11 variants suitable for Agena genotyping (sequenom maldi-tof technology) were tested in all samples included in the study. Variants were considered validated when they were found in the corresponding SCC and in none of the other samples, including the corresponding blood DNA. Six additional variants were selected and tested with ddPCR rare event detection assays in SCC, blood and muscle of the donor where they were discovered (see also Supplementary Tables 11 and 12).

Supplementary Table 6. INDEL classes

individual	clone n.	age	deletions 2-20 nt	deletions >20 nt	insertions 2-20 nt	insertions >20 nt
CES1	P2703_101	24	17	4	7	1
	P2703_102	24	16	1	7	2
	P2703_103	24	4	1	2	1
	P2703_104	24	24	3	9	1
	P2703_105	24	23	2	5	2
CES7	P2703_131	21	34	0	9	3
	P2703_132	21	20	7	10	0
	P2703_133	21	30	1	7	1
	P2703_134	21	17	2	6	3
	P2703_135	21	34	0	9	1
CES8	P5364_103	24	23	1	10	2
	P5364_104	24	32	2	8	1
	P5364_105	24	24	4	6	1
CES2	P2703_109	75	63	1	19	0
	P4206_203	75	53	0	16	0
CES3	P2703_111	63	50	6	9	2
	P2703_112	63	64	2	15	3
	P2703_113	63	64	5	10	3
	P2703_114	63	48	4	13	1
	P2703_115	63	48	2	9	1
CES4	P2703_116	67	51	1	12	2
	P2703_117	67	52	1	11	0
	P2703_118	67	54	1	11	4
	P2703_120	67	45	2	11	1
CES6	P2703_126	78	40	1	6	0
	P2703_127	78	55	5	10	4
	P2703_128	78	42	1	4	1
	P2703_129	78	81	4	20	4
	P2703_130	78	50	2	9	4

Number of indels/SCC according to different deletion and insertion classes.

Supplementary Table 7. Average number of somatic mutations in differentially expressed genes after muscle training.

Reference study	stimulus	n. genes diff expressed (gene set)	n. mutations hitting gene set in young	average no. mutations/ gene in young	n. mutations hitting gene set in old	average no. mutations/gene in old
4	aerobic exercise training	387	0	0.00000	3	0.00048
5	aerobic exercise training	100	0	0.00000	1	0.00063
6	resistance exercise training	661	2	0.00023	5	0.00047
7	resistance exercise training	512	0	0.00000	4	0.00049
8	endurance training	2624	6	0.00018	18	0.00043

Specific gene sets were tested for the occurrence of somatic mutations in young and old SCCs. Lists of differentially expressed genes after training were obtained from the published studies indicated in the first column.

Supplementary Table 8. Average number of somatic mutations in genes involved in pathways modulating the response to training

pathway	n. genes in pathway (gene set)	n. mutations hitting gene set in young	average no. mutations/ gene in young	n. mutations hitting gene set in old	average no. mutations/ gene in old
insulin mediated blood glucose clearence (Insulin-IRS-PI3K-TBC1D4-GLUT4 pathway)	407	0	0.00000	2	0.00031
generation of new mitochondria (AMPK-PGC1a pathway)	103	0	0.00000	1	0.00061
de novo ribosome biogenesis	312	2	0.00049	1	0.00020
cellular hypertrophy in SkM	125	0	0.00000	1	0.00050
activation and recruitment of resident stem cells in muscle	495	0	0.00000	8	0.00101

Specific gene sets were tested for the occurrence of somatic mutations in young and old SCCs. Lists of the genes involved in the pathway that modulates the response to training were obtained from curated gene sets from GO, KEGG, Biocarta and Reactome. Curated databases were accessed through the GSEA (Molecular Signature Database, Broad Institute). “Insulin,” “AMPK” and “ribosome” pathways are not specific to the skeletal muscle. Conversely, all curated gene sets included in “hypertrophy” include the words “skeletal muscle” in their description, and all the sets in “activation and recruitment of resident stem cells” include the word “muscle”.

Supplementary Table 9. Genes differentially expressed with age

Reference study	stimulus	n. genes diff expressed (gene set)	n. mutations hitting gene set in young	average no. mutations/ gene in young	n. mutations hitting gene set in old	average no. mutations/gene in old
[6]	SkM aging	49	0	0.00000	1	0.00128
[7]	SkM and brain aging	542	0	0.00000	6	0.00069
[9]	SkM aging	148	2	0.00104	5	0.00211
[10]	SkM aging	957	0	0	10	0.00065
GenAge	All organs aging	305	0	0	4	0.00082

Specific gene sets were tested for the occurrence of somatic mutations in young and old SCCs. Lists of differentially expressed genes during aging were obtained from the published studies indicated in the first column and were all specifically related to the skeletal muscle, except for GenAge, which corresponds to the human dataset obtained from the aging gene database (http://genomics.senescence.info/genes/).

Supplementary Table 10. Average number of somatic mutations found in old SCCs in exons, introns and flanking regions of genes involved in muscle diseases.

	Average no. somatic mutations/genome			
**Gene symbol**	**Exons**	**Introns**	**Upstream**	**Downstream**
ACVR1	0	0.125	0	0
AGL	0	0.0625	0	0
ANO5	0	0.0625	0	0
CACNA1A	0	0.125	0	0
CHAT	0	0.0625	0.0625	0
CHRNA1	0	0	0	0.0625
CLCN1	0	0.0625	0	0
CNTN1	0	0.125	0	0
COL6A3	0	0	0.0625	0
DAG1	0	0.125	0	0
DNM2	0	0.0625	0	0
DYSF	0	0	0.0625	0
ETFDH	0	0.0625	0	0
GBE1	0	0.125	0	0
GYS1	0	0.0625	0	0
HSPG2	0.0625	0	0	0
KCNA1	0	0.0625	0	0
KCNQ1	0	0.0625	0	0
LAMA2	0	0.0625	0	0
MYH8	0	0.0625	0	0
MYOT	0	0.0625	0	0
PNPLA8	0	0.1875	0	0
POLG2	0	0.0625	0	0
PREPL	0	0.0625	0	0
PRKAG2	0	0	0.0625	0
PTRF	0	0.0625	0	0
SGCD	0	0.375	0	0
SGCG	0	0.125	0	0
STIM1	0	0.125	0	0
SYNE1	0	0.0625	0	0
SYNE2	0.0625	0	0	0
TCAP	0	0	0	0.0625
TTN	0.0625	0.0625	0	0

The average number of mutations in different gene-related regions (exons, introns, 5 kb upstream and 5 kb downstream) in old SCCs (N=16) is shown. List of tested genes is shown in supplementary note 1. All genes present in supplementary note 1 and not mentioned in here did not show any somatic variant in old SCCs. Genes on chromosome X (*ALG13, DMD, EMD, FHL1, LAMP2, MTM1, PGK1, PHKA1, VMA21*) could not be addressed, as only autosomic variants were analyzed in the study (see methods).

Supplementary Table 11. List of variants tested by ddPCR.

gene	*individual*	*clone*	*variant*	*variant type*	*deleterious impact on protein**
FN1	CES4	P2703_120	chr2:216272892 C>T	synonym.	yes
HSPG2	CES6	P2703_127	chr1:22174499 G>A	missense	yes
FNDC1	CES6	P2703_129	chr6:159682281 T>A	missense	yes
TTN	CES3	P2703_111	chr2:179560728 G>A	synonym.	no
RAP1GDS1	CES3	P2703_113	chr4:99338610 C>T	missense	yes
CACNA2D2	CES3	P2703_113	chr3:50426879 C>T	intron	yes

*mutations were classified as deleterious when they were scored deleterious by at least one program (PolyPhen, SIFT, Mutation Taster 2 [11], CADD v.1.1 [12])

Supplementary Table 12. Fractional abundance of variants tested by ddPCR in all tested samples.

sample	fractional abundance of the mutant allele (%)	total number of detected mutant alleles	total number of analyzed alleles
HSPG2 c.7825C>T			
CES6 P2703_127	49.94	7700	15420
CES6 muscle	1.29	928	72088
control muscle	0.06	30	48110
CES6 blood	0.10	54	56394
CES8 blood	0.05	19.6	39199.6
CES6 D6.2	0	0	2010
CES6 H8	0	0	2078
CES6 B4	0	0	2258
CES6 C9	0	0	2424
CES6 A12.2	0	0	2083.6
CES6 F11	0	0	2620
CES6 B5	0	0	2257.6
CES6 D11	0	0	2161.8
CES6 E6	0	0	3060
CES6 E11	0	0	3389.4
CES6 F10	0	0	3761.6
CES6 G2	0	0	2035.6
CES6 B11.2	0	0	2299.4
CES6 muscle cDNA	1.95	1038	53198
CES6 muscle noRT cDNA	0	0	0
CES8 muscle cDNA	0	0	57442.8
control CD56+ population	0.02	12.4	59352.4
control CD56+ population noRT cDNA	0	0	0
CES6 CD56+ population	0.04	16.2	39396.2
CES6 CD56+ population cDNA	0	0	60
TTN c.31071C>A			
CES3 P2703_111	49.54	26820	54140
CES3 muscle	0.03	23.4	91623.4
control muscle	0.02	18.2	81878.2
CES3 blood	0.04	21.2	50501.2
CES8 blood	0.04	18.2	43878.2
CES3 CD56+ population	0.02	9.4	44249.4
FNDC1 c.5234T>A			

CES6 P2703_129	49.3	4940	10020
CES6 muscle	0.01	5.8	88065.8
control muscle	0	0	48483.2
CES6 blood	0	0	65023
CES8 blood	0	0	51441.6
CES6 CD56+ population	0	0	39821.6
FN1 c.2457G>A			
CES4 P2703_120	49.32	5760	11680
CES4 muscle	0.03	8	28188
control muscle	0.02	10.2	41630.2
CES4 blood	0.03	9.2	32209.2
CES8 blood	0.02	8	48628
CES4 CD56+ population cDNA [illegible]	0	0	8840
RAP1GDS1 c.1003C>T			
CES3 P2703_113	48.94	9740	19900
CES3 muscle	0.02	8.2	42668.2
control muscle	0.02	12.2	60992.2
CES3 blood	0.03	10.2	31530.2
CES8 blood	0.02	12.2	54172.2
CES3 CD56+ population	0.02	6.6	42286.6
CACNA2D2			
CES3 P2703_113	48.7	9380	19260
CES3 muscle	0.02	8.2	39368.2
control muscle	0.02	9.2	50809.2
CES3 blood	0.01	4.2	31444.2
CES8 blood	0	0	41641.2
CES3 CD56+ population	0.02	6.4	41166.4

Supplementary References

1. Blokzijl, F., *et al.* Tissue-specific mutation accumulation in human adult stem cells during life. *Nature* 538, 260-264 (2016).
2. Nik-Zainal, S., *et al.* Mutational processes molding the genomes of 21 breast cancers. *Cell* 149, 979-993 (2012).
3. Abyzov, A., *et al.* One thousand somatic SNVs per skin fibroblast cell set baseline of mosaic mutational load with patterns that suggest proliferative origin. *Genome Res* (2017).
4. Timmons, J.A., *et al.* Human muscle gene expression responses to endurance training provide a novel perspective on Duchenne muscular dystrophy. *FASEB J* 19, 750-760 (2005).
5. Timmons, J.A., *et al.* Using molecular classification to predict gains in maximal aerobic capacity following endurance exercise training in humans. *J Appl Physiol (1985)* 108, 1487-1496 (2010).
6. Raue, U., *et al.* Transcriptome signature of resistance exercise adaptations: mixed muscle and fiber type specific profiles in young and old adults. *J Appl Physiol (1985)* 112, 1625-1636 (2012).
7. Phillips, B.E., *et al.* Molecular networks of human muscle adaptation to exercise and age. *PLoS Genet* 9, e1003389 (2013).
8. Lindholm, M.E., *et al.* The Impact of Endurance Training on Human Skeletal Muscle Memory, Global Isoform Expression and Novel Transcripts. *PLoS Genet* 12, e1006294 (2016).
9. Sood, S., *et al.* A novel multi-tissue RNA diagnostic of healthy ageing relates to cognitive health status. *Genome Biol* 16, 185 (2015).
10. Su, J., *et al.* A novel atlas of gene expression in human skeletal muscle reveals molecular changes associated with aging. *Skelet Muscle* 5, 35 (2015).
11. Schwarz, J.M., Cooper, D.N., Schuelke, M. & Seelow, D. MutationTaster2: mutation prediction for the deep-sequencing age. *Nat Methods* 11, 361-362 (2014).
12. Kircher, M., *et al.* A general framework for estimating the relative pathogenicity of human genetic variants. *Nat Genet* 46, 310-315 (2014).
