# Supplementary material for: Somatic mutagenesis in satellite cells associates with human skeletal muscle aging
Source: Nat Commun. 2018 Feb 23;9:800. doi: 10.1038/s41467-018-03244-6 (PMC5824957; doi:10.1038/s41467-018-03244-6)
Supplement: Supplementary file 3 — Description of Additional Supplementary Files [file 41467_2018_3244_MOESM3_ESM.pdf]

Description of Additional Supplementary Files

File Name: Supplementary Data 1
Description: Filtered somatic mutations in young human satellite cells

File Name: Supplementary Data 2
Description: Filtered somatic mutations in old human satellite cells

File Name: Supplementary Data 3
Description: Filtered somatic mutations in long-cultured human satellite cells
